# SIMOA Diagnostics on Alzheimer’s Disease and Frontotemporal Dementia

**DOI:** 10.3390/biomedicines12061253

**Published:** 2024-06-04

**Authors:** Athanasia Chatziefstathiou, Sezgi Canaslan, Eirini Kanata, Kostas Vekrellis, Vasilios C. Constantinides, George P. Paraskevas, Elisabeth Kapaki, Matthias Schmitz, Inga Zerr, Konstantinos Xanthopoulos, Theodoros Sklaviadis, Dimitra Dafou

**Affiliations:** 1 Department of Genetics, Development, and Molecular Biology, School of Biology, Aristotle University of Thessaloniki, 54124 Thessaloniki, Greece; chatziea@bio.auth.gr; 2 Department of Neurology, German Center for Neurodegenerative Diseases (DZNE), University Medicine Göttingen, 37075 Göttingen, Germany; sezgi.canaslan@med.uni-goettingen.de (S.C.); matthias.schmitz@med.uni-goettingen.de (M.S.); ingazerr@med.uni-goettingen.de (I.Z.); 3 Neurodegenerative Diseases Research Group, Department of Pharmacy, School of Health Sciences, Aristotle University of Thessaloniki, 54124 Thessaloniki, Greece; ekanata@pharm.auth.gr (E.K.); xantho@pharm.auth.gr (K.X.); sklaviad@pharm.auth.gr (T.S.); 4 Center of Basic Research, Biomedical Research Foundation of the Academy of Athens, 11527 Athens, Greece; vekrellis@bioacademy.gr; 5 Neurochemistry and Biological Markers Unit, First Department of Neurology, School of Medicine, National and Kapodistrian University of Athens, Eginition Hospital, 11528 Athens, Greece; vassilis.kon@hotmail.com (V.C.C.); ekapaki@med.uoa.gr (E.K.); 6 Second Department of Neurology, School of Medicine, National and Kapodistrian University of Athens, “Attikon” University General Hospital, 12462 Athens, Greece; geoprskvs44@gmail.com

**Keywords:** SIMOA platform, cerebrospinal fluid, neurodegenerative diseases, biomarkers, multiplex, AD, FTD, sPLS-DA, dementias

## Abstract

Background: Accurate diagnosis of Alzheimer’s disease (AD) and frontotemporal dementia (FTD) represents a health issue due to the absence of disease traits. We assessed the performance of a SIMOA panel in cerebrospinal fluid (CSF) from 43 AD and 33 FTD patients with 60 matching Control subjects in combination with demographic–clinical characteristics. Methods: 136 subjects (AD: *n* = 43, FTD: *n* = 33, Controls: *n* = 60) participated. Single-molecule array (SIMOA), glial fibrillary acidic protein (GFAP), neurofilament light (NfL), TAU, and ubiquitin carboxy-terminal hydrolase L1 (UCH-L1) in CSF were analyzed with a multiplex neuro 4plex kit. Receiver operating characteristic (ROC) curve analysis compared area under the curve (AUC), while the principal of the sparse partial least squares discriminant analysis (sPLS-DA) was used with the intent to strengthen the identification of confident disease clusters. Results: CSF exhibited increased levels of all SIMOA biomarkers in AD compared to Controls (AUCs: 0.71, 0.86, 0.92, and 0.94, respectively). Similar patterns were observed in FTD with NfL, TAU, and UCH-L1 (AUCs: 0.85, 0.72, and 0.91). sPLS-DA revealed two components explaining 19% and 9% of dataset variation. Conclusions: CSF data provide high diagnostic accuracy among AD, FTD, and Control discrimination. Subgroups of demographic–clinical characteristics and biomarker concentration highlighted the potential of combining different kinds of data for successful and more efficient cohort clustering.

## 1. Introduction

The two most common causes of dementia in elderly people are Alzheimer’s disease (AD) and frontotemporal dementia (FTD). Memory and cognitive decline are among the main features of AD, while FTD is a highly heterogeneous disorder. The manifestations of FTD include behavioral alterations, difficulty with language, motor symptoms, and other mental health conditions, such as personality and behavioral changes [1,2]. AD’s pathological hallmarks include the deposition of amyloid beta (Ab) plaques in the brain’s neocortex with neurofibrillary tangle compositions, while in FTD, most patients show a degeneration of the frontal and temporal lobes, including paralimbic areas [2,3,4]. The biochemical profile of AD in cerebrospinal fluid (CSF) has been established for over 20 years, following the seminal works of Vandermeeren et al. regarding total-TAU protein [5], Motter et al. regarding Ab with 42 amino acids [6], and Ishiguro et al. regarding phosphorylated TAU protein (ph-TAU) [7].

Diagnosis of dementia represents a challenging public health issue as it can be particularly difficult in cases where disease-specific traits are absent. Incorrect or delayed diagnosis between AD and FTD may result in serious consequences in a patient’s treatment efficacy, pharmacologic accuracy, therapeutic interventions, and disease management [1]. Since these disorders are challenging to diagnose clinically, various biomarkers of underlying pathophysiology have been used to increase diagnostic precision and play a crucial role in research and clinical trials [8,9]. For clinical trials of disease-modifying treatments in particular, biomarkers which accurately reflect the underlying pathology in pre- or oligosymptomatic stages of different dementias are pivotal for correct patient stratification. A promising biomarker must reflect not only disease development and/or progression but also disease severity and treatment efficiency [10,11]. An optimal discriminatory biomarker for neurodegenerative disorders (NDs) would exhibit high sensitivity and specificity, should be reproducible, preferentially non-invasive, and easy to measure, utilizing a cost-effective platform set-up [12].

Well-studied biomarkers such as Ab40-42 [13,14,15], TAU, or ph-TAU [16] are established clinical diagnostic markers for AD; however, they are not highly predictive of memory and cognitive impairments. Ongoing efforts focus on standardizing protocols and establishing criteria for the proper use of biomarkers in the diagnosis of the disease, intending to distinguish one neurodegenerative disease from another [2]. Other non-disease-specific biomarkers, reflecting neurodegeneration in general, could be utilized in combination with Ab and TAU, with the potential to enhance differential diagnosis in dementias [17]. Thus, apart from already-established biomarkers, recent studies have identified new candidates for NDs discrimination, such as the neurofilament light (NfL) protein, glial fibrillary acidic protein (GFAP), and ubiquitin carboxy-terminal hydrolase L1 (UCH-L1) [3,18,19,20,21].

The selection of an appropriate biomarker panel is of paramount importance in differential diagnosis of AD and FTD. Recent studies have focused on the use of UCH-L1, NfL, and TAU, which localize in different areas of a neuron, as potential biomarkers [22]. In addition, the potential of GFAP in clinical diagnosis has been highlighted after a GFAP–UCH-L1 blood test was authorized by the Food and Drug Administration (FDA) for use in mild traumatic brain injury (TBI) [10]. GFAP, an astrocyte-specific marker, is a filament of mature astrocytes, distributed in the white and gray matter, the cerebellum, and the subventricular and subgranular zones [23]. GFAP seems to be essential for the activation of astroglial cells (astrogliosis) that occurs after central nervous system (CNS) trauma, as well as during neurodegeneration [24]. In line with this, in AD brains, GFAP levels are higher in regions around Ab plaques with elevated TAU accumulation [12]. Recently, preliminary evidence categorized GFAP as a potential plasma biomarker for AD, rather than CSF, as it showed better performance when detected in plasma samples [25]. It has also been suggested that GFAP and UCH-L1 form a biomarker duplex representing the two most dominant cell types in the brain, astrocytes, and neurons [26]. UCH-L1, a highly abundant neuron-specific cytoplasmic enzyme, modifies the activity of the ubiquitin proteasome system (UPS) by functioning as a deubiquitinating hydrolase, ubiquitin ligase, and a monoubiquitin stabilizer. The presence of UCH-L1, along with ubiquitin, in Ab plaques and neurofibrillary tangles in AD brains is an additional proof that UPS is dysfunctional in AD pathology. Increased levels of CSF ubiquitin and UCH-L1 have been reported in several previous studies in AD patients, suggesting that CSF UCH-L1 may distinguish AD patients from Control subjects with high diagnostic accuracy [27]. However, even though UCH-L1 is one of the few TBI biomarker candidates identified, based on recent proteomic studies, the diagnostic accuracy of CSF UCH-L1 in discriminating AD from other NDs should be further investigated [26]. Another biomarker of great importance is NfL. Neurofilaments are cytoskeletal neuronal proteins composed of three subunits: light, medium, and heavy chains. They are essential for the structural stability of neurons and are highly expressed in myelinated axons [1]. NfL is released after neuronal damage both in the CSF and bloodstream, and its elevated concentration in CSF has been described in many NDs [23]. Although NfL has not been categorized as a diagnostic biomarker for a specific ND, it has the potential to become a useful tool; in combination with other markers, it could contribute to monitoring ND progression [1]. In comparison, TAU protein, an already-established biomarker for AD, is expressed in a variety of mammalian tissues but predominantly in CNS neurons. TAU, as a microtubule-associated protein, is important to neuronal structure, function, and axonal processes, as it is primarily found within axons [28]. TAU is a naturally unfolded, highly soluble protein that interacts with tubulin, promoting its assembly into microtubules, thus contributing to the stabilization of their structure [29].

The clinical implementation of these biomarkers (either as individual markers or in combination) requires a detailed assessment of their diagnostic value. Additionally, considering the prevalence of mixed brain pathologies in the dementia spectrum, the impact of coexisting and overlapping comorbidities should be taken into consideration when evaluating potential biomarker’s accuracy [25].

To date, the clinical diagnosis of ND patients, including AD and FTD, has mostly been based on imaging and biomarker measurements in CSF or blood (serum or plasma). Methods already used are the enzyme-linked immunosorbent assay (ELISA) [30] and the real-time quaking-induced conversion assay (RT-QuIC) [31]. Τhis combinatory diagnostic protocol currently in use could be improved by novel detection methods like electrochemiluminescence immunoassays (ECLIA) and immunomagnetic reduction (IMR) [30], or by the utilization of biomarker panels like Luminex xMAP [32], NeuroToolKit [17], and single-molecule array (SIMOA) [33]. Limitations associated with current and widely used analytical methods refer to difficult maintenance in large cohort studies, relatively high detection limits, and the requirement of high sample volumes. These restrictions hamper the study of potential novel biomarkers, especially in plasma, where levels are usually lower compared to CSF.

SIMOA, a state-of-the-art technology, identifies and quantifies immunocomplexes bound to dye-encoding magnetic beads (using different capture and detector antibodies) sealed in arrays of femtoliter-volume microwells. SIMOA requires low sample volumes and allows for multiplexing analysis, which has been used for blood and CSF biomarker detection [33,34,35]. This advanced and highly sensitive technique represents a valid tool for new biomarkers detection, giving new perspectives with promising future to a whole new research field [25,36].

In this study, we aimed to assess the diagnostic performance (sensitivity, specificity and optimal cut-off values) of a multiplex panel of four biomarkers—GFAP, NfL, TAU, and UCH-L1—in CSF samples across a clinical cohort of 43 AD and 33 FTD patients and 60 Control subjects using the SIMOA platform. Our subsequent goal was to further stratify the samples through the identification of different cohort clusters based on combinations of biomarkers (new and already established) and demographic–clinical characteristics. More specifically, we applied the classification performance of the principal of the sparse partial least squares discriminant analysis (sPLS-DA) model in our dimensional dataset to identify the most relevant variables to succeed in meaningful cohort clustering.

## 2. Materials and Methods

### 2.1. Ethical Considerations

All patients or their next of kin (in cases of compromised mental capacity) provided written informed consent for participation in this study. The study was approved by the Scientific and Ethics Committee of Eginition Hospital, by the Local Ethics Committee of the University of Medicine, Göttingen, No. 19/11/09, “Liquormarker als Prädiktoren für die Entwicklung einer Demenz bei Patienten mit Morbus Parkinson, Demenz mit Lewy Körperchen und Morbus Alzheimer”, and by the Ethics Committee of the University Medicine Göttingen, No. 13/11/12 “LIX—Liquormarker zur Frühdiagnose und Krankheitsprogression bei Patienten mit Parkinson-syndromen und Motoneuronerkrankungen” and performed in accordance with the guidelines of the 1964 Declaration of Helsinki.

### 2.2. Patients

The medical files of all patients with available data on CSF biomarkers (such as Ab42, Ab40, total-TAU, and ph-TAU-181), admitted from 2011 to 2021 to the “Neurodegenerative Disorders and Epilepsy” Ward of Eginition Hospital, Athens, Greece, were retrospectively reviewed. All patients included were consecutively admitted to our clinic. All patients underwent a thorough diagnostic work-up, including a detailed medical history, neurological examination, neuropsychological assessment, including the mini mental state examination (MMSE—a test of global cognitive status) [37], frontal assessment battery (FAB—a test of frontal executive function) [38], 5 word recall test (a test of memory) [39], and CLOX test (a test of visuospatial–visuoconstructive function) [40]. Based on clinical data, a final diagnosis was established by three neurologists with extensive experience in cognitive disorders (E.K., G.P.P., V.C.C.), blinded to the CSF biomarker profile. Only subjects with a consensus among the three neurologists regarding the clinical diagnosis were included. Subjects were included if they fulfilled the established diagnostic criteria for AD [39] and FTD [40] (AD: *n* = 43; FTD: *n* = 33). The Control group consisted of otherwise healthy subjects, with no comorbidities, undergoing knee or hip joint surgery or hernia repair under spinal anesthesia. These subjects had a negative history of cognitive or behavioral/psychiatric disorders and no clinical evidence of any major disease (healthy Controls: *n* = 12) or of diagnosis of non-primary neurodegenerative neurological and psychiatric conditions according to the acknowledged standard of neurological, clinical, and para-clinical findings based on the Internation classification of diseases, tenth revision (ICD-10) definition cases; they were without cognitive impairment or dementia at the time of sampling (non-neurodegenerative neurological Controls: *n* = 48). All Control subjects had normal scores on neuropsychological testing (MMSE and FAB). Blood-contaminated CSF samples were excluded from the study [37,38].

### 2.3. CSF Sampling and Biomarker Measurements

All patients underwent lumbar puncture at 10–11 a.m. after overnight fasting based on standard operating procedures in accordance with recommendations to standardize preanalytical confounding factors in AD CSF biomarkers [41].

GFAP, UCH-L1, NfL, and TAU concentrations in the CSF were determined using a commercial assay kit [Neuro 4 plex, Quanterix, Billerica, MA, USA (Product number: 103345)] that had already been optimized for certain marker proteins and measured in the SIMOA-SR-X instrument (Quanterix, Billerica, MA, USA). Analysis was performed according to the manufacturer instructions. CSF samples were diluted 40× to a total volume of 100 μL. Two internal Controls with a defined protein concentration were included in the assay, and data were subjected to further analysis only when both internal assay Controls were within the expected range (less than 10% variation).

CSF samples were vortexed for 10–20 s and centrifuged for 5 min at 10,000 rpm to remove any impurities before use. Initially, each sample was analyzed in duplicates, displaying statistically insignificant intra-assay variation, allowing us to proceed with single measurements due to low sample volumes. All samples were anonymized and analyzed blindly and randomly by the experimenter.

### 2.4. Statistical Analysis

The normality of distribution was assessed via Shapiro–Wilk’s test. Comparison of demographic/clinical data was performed via χ^2^ test, ordinary one-way ANOVA, or *t*-test (Mann–Whitney test or unpaired *t*-test) as appropriate. The spread of value comparisons of CSF biomarkers in the study groups was performed via unpaired ANOVA (Kruskal–Wallis test and Dunn’s multiple comparison test) as appropriate.

Receiver operating characteristic (ROC) curve analysis [calculating the area under the curve (AUC) ± standard error (SE) and its 95% confidence interval (CI), alongside the cut-off points and specificity, sensitivity, and 95% CI and Youden Index (YI) of the optimal cut-off points] was performed to compare the diagnostic accuracy of all four CSF biomarkers among patients with AD and FTD and the Control group. All correlation studies were computed using the non-parametric Spearman’s correlation test (two-tailed) with a CI of 95%. For statistical analysis, the GraphPad Prism 8.0.2 (263) software (GraphPad, San Diego, CA, USA) was used. *p*-values below 0.05 were considered statistically significant [41,42].

The principal of sPLS-DA was used to create possible cohort clusters based on different types of data. R software (R foundation for statistical computing 4.2.0.1 Rtools, Vienna, Austria) and R studio were used for sPLS-DA analysis. We used the MixOmics package, which is an R toolkit dedicated to the exploration and integration of biological datasets, with a specific focus on variable selection. sPLS-DA is a supervised, multivariate analysis approach to identify a set of variables (in this case, biomarkers and demographic–clinical data) accounting for the greatest variation present in the dataset. The usage of this supervised exploratory approach enables the assessment of the generalization properties of the model and allows for the selection of suitable variables for the outcome status prediction of the patients. In our analysis, it allows the use of additional patients’ data from different clinical tests (i.e., CSF ELISA, CSF SIMOA, and plasma SIMOA) without direct comparisons with matching Controls. The inclusion of such data increases the strength of the analysis and of the discriminatory potential of our CSF SIMOA markers in AD and FTD [43].

In this context only, we analyzed 12 biomarkers in total, the SIMOA-measured biomarkers (GFAP, NfL, TAU, and UCH-L1) in CSF of 43 AD, 33 FTD, and 60 Control patients, as described earlier, as well as additional data (Supplementary File, Table S1) of the same panel of four biomarkers in plasma and data from ELISA-measured biomarkers (Ab42, Ab40, total-TAU, ph-TAU) in CSF of the same patient cohort [AD (*n* = 43) and FTD (*n* = 33) patients]. Moreover, we used 9 demographic–clinical characteristics (gender, age, disease duration, MMSE, FAB, 5 word recall, CLOX2, Sheltens L, Sheltens R). sPLS-DA facilitated the ability to combine different types of data to strengthen our original hypothesis regarding the increased discriminatory potential of our four CSF SIMOA biomarkers in AD and FTD. Loading plots were generated to visualize the variables responsible for clustering.

## 3. Results

### 3.1. Clinical and Demographic Data

In total, 136 subjects were included in this study (AD: 43 patients; FTD: 33 patients; Control: 60 subjects). Study groups did not differ in sex (*p* = 0.4102); however, they differed in age (*p* < 0.0001). Demographic, clinical data, and SIMOA-measured biomarkers (GFAP, NfL, TAU, and UCH-L1) in CSF of AD, FTD, and Controls are summarized in Table 1.

### 3.2. Assessement of CSF GFAP, NfL, TAU, and UCH-L1 Diagnostic Accuracy

**Figure 1:**
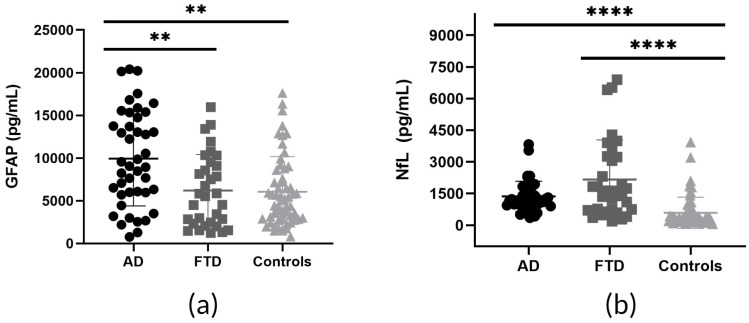

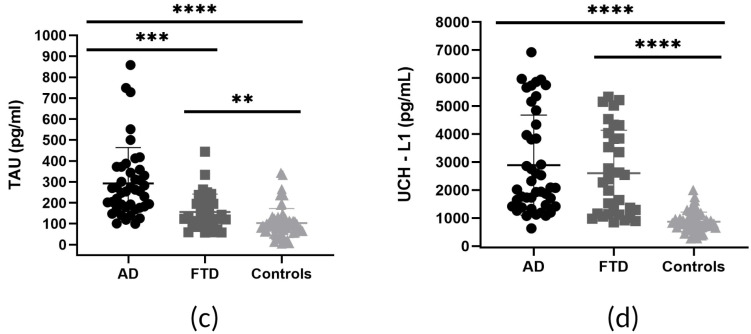
Glial fibrillary acidic protein (GFAP), neurofilament light chain (NfL), TAU, and ubiquitin carboxy-terminal hydrolase L1 (UCH-L1) levels in cerebrospinal fluid (CSF) of Alzheimer’s disease (AD), Frontotemporal dementia (FTD), and Control subjects. (**a**) A statistically significant increase in GFAP levels was observed in AD compared to Controls (Dunn’s multiple comparison test *p* = 0.0012) and FTD cases (Dunn’s multiple comparison test *p* = 0.0078). (**b**) NfL levels were significantly increased in AD compared to Controls (Dunn’s multiple comparison test *p* < 0.0001) and similarly in FTD cases compared to Controls (Dunn’s multiple comparison test *p* < 0.0001). (**c**) TAU levels were significantly increased in AD patients relative to Controls (Dunn’s multiple comparison test *p* < 0.0001) or FTD (Dunn’s multiple comparison test *p* = 0.0003). In addition, TAU levels were significantly increased when the FTD–Control comparison was considered (Dunn’s multiple comparison test *p* = 0.0097). (**d**) UCH-L1 levels were increased in AD patients relative to Controls (Dunn’s multiple comparison test *p* < 0.0001), as well as in FTD cases compared to Controls (Dunn’s multiple comparison test *p* < 0.0001). Scatter plots display individual values, means, and Standard Deviations (SDs). Statistical significance is denoted as follows: **** *p*-value < 0.0001; *** *p*-value < 0.001; and ** *p*-value < 0.01.

CSF GFAP, NfL, TAU, and UCH-L1 levels were determined by SIMOA in AD, FTD, and Control samples. Our ANOVA/non-parametric Kruskal–Wallis test analysis identified increased levels of all tested biomarkers in AD and FTD cases compared to Controls. Specifically, clear differences in the levels of all four biomarkers were detected in AD cases relative to Controls. A similar pattern was evident in the FTD samples, where the levels of the three biomarkers (NfL, TAU, and UCH-L1) were statistically significantly different from those of the Controls. Interestingly, differences in GFAP and TAU levels were statistically significant between AD and FTD, suggesting their potential use in differential diagnosis (Figure 1).

**Figure 2:**
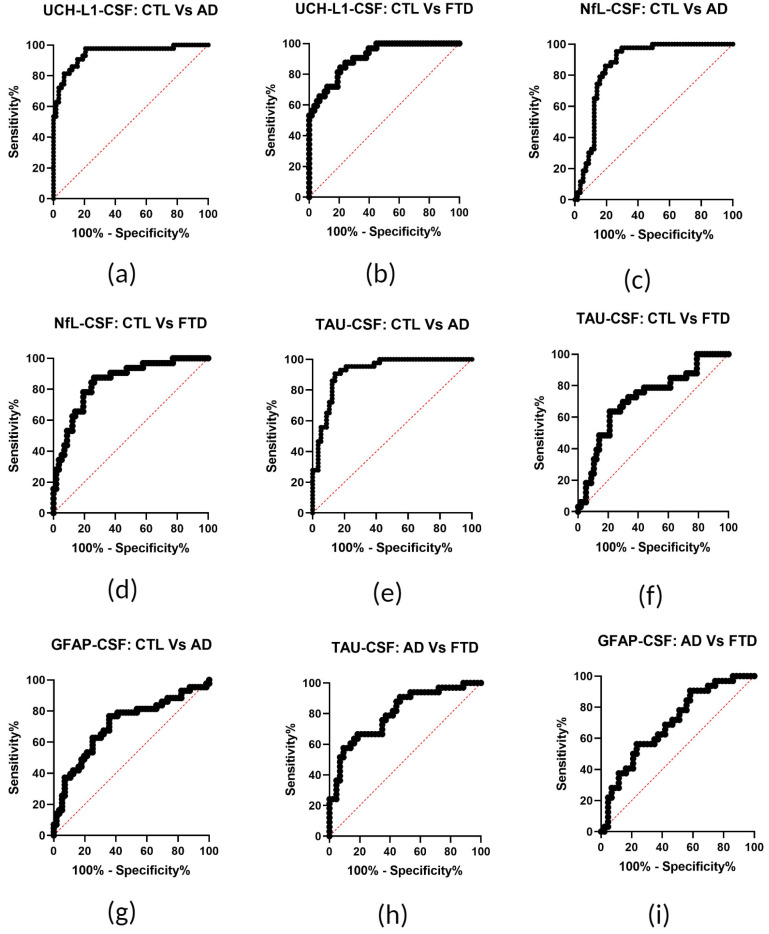
Receiver operating characteristic (ROC) curve analysis of CSF GFAP, NfL, TAU, and UCH-L1 levels from patients with AD, FTD, and Controls (CTL). (**a**,**b**) The diagnostic accuracy of CSF UCH-L1 was assessed for the discrimination of patients with AD and FTD from CTL. AUC values of 0.94 ± 0.02 SE and 0.91 ± 0.03 SE, respectively, indicated excellent diagnostic accuracy. (**c**,**d**) For the discrimination of AD and FTD patients from CTL, the diagnostic accuracy of CSF NfL was assessed with AUC values of 0.86 ± 0.04 SE and 0.85 ± 0.04 SE, respectively, indicating a good diagnostic accuracy. (**e**,**f**) The diagnostic accuracy of CSF TAU was assessed for the discrimination of AD and FTD patients from CTL, with AUC values of 0.92 ± 0.03 SE and 0.72 ± 0.06 SE, respectively, indicating a very good diagnostic accuracy. (**g**) The diagnostic accuracy of CSF GFAP in the discrimination of AD patients from CTL with an AUC value of 0.71 ± 0.05 SE indicated a fair diagnostic accuracy. (**h**) The diagnostic accuracy of CSF TAU in the discrimination of AD patients from FTD patients was assessed, with an AUC value of 0.8 ± 0.05 SE indicating a good diagnostic accuracy. (**i**) The diagnostic accuracy of CSF GFAP was assessed with AUC values for the discrimination of AD patients from FTD. The AUC value of 0.7 ± 0.06 SE indicated a fair diagnostic accuracy.

ROC curve and AUC analysis (GraphPad Prism 8.0.2 (263)) were used to assess the diagnostic accuracy of the tested biomarkers. UCH-L1 and TAU showed excellent discrimination efficiency between AD cases and Controls (AUC: 0.94 ± 0.02 SE, 95% CI 0.90–1, *p* < 0.0001; AUC: 0.92 ± 0.03 SE, 95% CI 0.87–0.97, *p* < 0.0001, respectively). A good differentiation between AD and Controls was also achieved by NfL (AUC: 0.86 ± 0.04 SE, 95% CI 0.79–0.94, *p* < 0.0001), while GFAP performed fairly (AUC: 0.71 ± 0.05 SE, 0.60–0.82 95% CI, *p* = 0.0003). UCH-L1 showed excellent performance in the differentiation between FTD cases and Controls (AUC: 0.91 ± 0.03 SE, 95% CI 0.85–0.97, *p* < 0.0001), followed by NfL (AUC: 0.85 ± 0.04 SE, 95% CI 0.77–0.93, *p* < 0.0001), in contrast to TAU, which displayed reduced discrimination ability (AUC: 0.72 ± 0.06 SE, 95% CI 0.60–0.83, *p* = 0.0006). Considering the differential diagnosis between AD and FTD, a good discrimination performance was detected for TAU (AUC: 0.80 ± 0.05 SE, 95% CI 0.70–0.90, *p* < 0.0001) in contrast to GFAP, which did not perform as well (AUC: 0.7 ± 0.06 SE, 95% CI 0.58–0.82, *p* = 0.0036) (Figure 2).

Aiming to calculate an optimal diagnostic cut off for AD and FTD diagnosis from the ROC curve analysis of Figure 2, we calculated the YI. We propose for CSF UCH-L1 a cut off >1260 pg/mL; for CSF NfL, a cut off >688.8 pg/mL; for CSF TAU, a cut off >137.0 pg/mL; and finally, for CSF GFAP, a cut off >5942 pg/mL as sufficient to distinguish between AD and Controls. Moreover, we propose for CSF UCH-L1 a cut off >1052 pg/mL; for CSF NfL, a cut off >463.9 pg/mL; and finally, for CSF TAU, a cut off >111.3 pg/mL as sufficient to distinguish between FTD and Controls (Table 2).

### 3.3. Sample Stratification Based on Biomarkers and Demographic–Clinical Data

**Figure 3:**
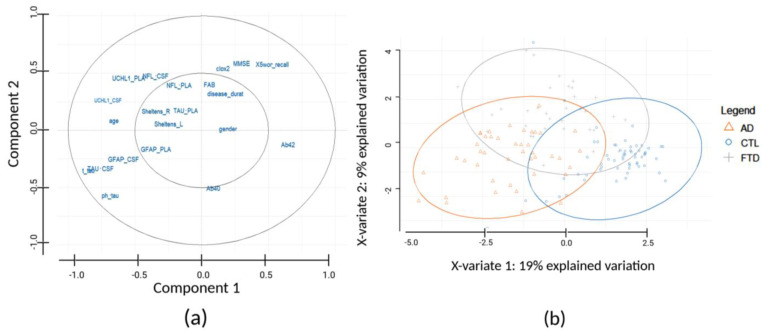
Identification of sample subgroups within the studied cohort by sPLS-DA using biomarkers and demographic–clinical characteristics. (**a**) Projection of the sPLS-DA variables (biomarkers and demographic–clinical data) on correlation circles, assessing the correlation of each variable in the space spanned by Component 1 and Component 2. Positively correlated variables group together, while negatively correlated variables are positioned on opposed quadrants. (**b**) The final individual plot with X-variates 1 and 2 in axes, indicating the amount of variation explained per component, i.e., 19% and 9% of variation, respectively, stratifying the samples in different cohort clusters.

For the identification of potential patient subgroups, sPLS-DA was applied, a statistical supervised classification method creating its own components based on the independent variable given, performing variable selection and classification to create distinct clusters. For this purpose, we included 12 biomarkers (supplementary date also used, Table S1) and 9 demographic–clinical characteristics, using the R MixOmics package in R studio (Figure 3). Every point in the variable plot (a) is used to visualize and assess the correlation of each variable to a selected set of two latent components. Positively correlated variables are grouped together, while negatively correlated variables are positioned on opposite sides on the plot (opposed quadrants). The distance between the different variables and the origin (0, 0) is a quality indicator on the factor map; i.e., variables that are away from the origin are the most well represented on the factor map. In our case (UCH-L1_CSF, UCH-L1_plasma, TAU_CSF, age, total-TAU, ph-TAU, GFAP_CSF and NfL_CSF), variables indicate strong negative correlation with Component 1, while only Ab42 variable showed strong positive correlation with Component 1. All the variables are located in the external circle. Regarding Component 2 variables (ph-TAU, Ab40, TAU_CSF, GFAP_CSF, GFAP_plasma and total-TAU), a strong negative correlation is indicated. UCH-L1_plasma, NfL_CSF, UCH-L1_CSF, 5 word recall, NfL_plasma, CLOX2, MMSE, TAU_plasma, disease duration, and age variables showed a strong positive correlation. In agreement with these results, an additional table (Table S2) for the correlation matrix of each variable with the latent Components 1 and 2 and a depicted heat map (Figure S1) of this correlation matrix are included in the Supplementary Files.

On the other hand, individual plot (b) statistical analysis revealed two components (referred as X-variate 1 and X-variate 2), explaining 19% and 9% of data variation, respectively. More specifically, plot (b) allows for the clustering and evaluation of the samples. In addition, the three sample groups (AD, FTD, and Controls) are presented in different colors. Samples classified on the right corner of plot (b) have relatively higher values for variables also classified on the right corner of plot (a), and samples classified on the left corner of the plot (b) have relatively higher values for variables also classified on the left of the plot (a). The subgroups of demographic–clinical characteristics and biomarker concentration data strengthen the performance of the analytic tool used, highlighting the advantage of combining different kinds of available data for efficient cohort clustering, without the direct comparison with matching Controls for every data point.

## 4. Discussion

Clinical diagnosis in the majority of NDs is based on clinical evaluation and imaging techniques like magnetic resonance imaging (MRI) and positron emission tomography (PET) scans. However, considering that (i) more than one ND may co-occur in the same patient; (ii) different NDs share common clinical characteristics; and (iii) neurodegenerative processes within the CNS present with a high degree of complexity and interindividual heterogeneity, there is a clear and unmet need of establishing appropriate biomarker panels that enable differential diagnosis [36,44,45]. The recent literature indicates that fluid and blood biomarker levels are detectable before symptom onset, highlighting the importance and utility of biomarkers in the diagnosis, progression, prognostication, and treatment efficiency of NDs [45,46]. CSF, as the organic liquid surrounding the brain and spinal cord, is expected to reflect neurodegenerative processes in the brain and is thus considered a valuable biological fluid for NDs biomarker studies [44,47]. The applied technology, SIMOA, allows for ultra-sensitive digital biomarker detection with the ability to quantify proteins in the lowest detectable levels (fg/mL) under the threshold of detection by traditional methods. Given its remarkable sensitivity in detecting molecules of very low concentration, SIMOA is preferably utilized for the identification of novel CSF molecules with potential use as biomarkers [33].

The primary goal of our study was to assess the differential diagnostic performance (sensitivity, specificity, and optimal cut-off values) of a panel of four biomarkers—GFAP, NfL, TAU, and UCH-L1—in CSF samples across a clinical cohort of 43 AD and 33 FTD patients and 60 Control subjects using the SIMOA platform.

Regarding the distinction of AD patients from the Control group, our study provided comparable diagnostic accuracy in our cohort. We identified increased levels of all tested CSF biomarkers (GFAP, NfL, TAU, and UCH-L1) in AD cases compared to Controls. In agreement with our data, most relevant studies in the literature showed a similar detection range for NfL and GFAP. According to neuropathological data, a close spatial association between Ab plaques and reactive astrocytes has been demonstrated. Together with microglia, these cells may initiate a pro-inflammatory cascade that ultimately leads to neurodegeneration. GFAP, a cytoskeletal component of astrocytes, has the potential to serve as a valuable biomarker of astrocytic activation and proliferation during neurodegenerative processes in AD pathology. Regarding NfL, it serves as a relatively non-specific biomarker for neurodegeneration as it is released due to axonal damage across various neurological disorders [48,49]. However, our ROC curve analyses highlighted an excellent diagnostic performance for TAU and UCH-L1 (AUC values of 0.92 and 0.94, respectively). In line with a previous study, UCH-L1 has been reported in AD pathology, with the capacity to distinguish AD patients form Control subjects with high diagnostic accuracy. Moreover, there has been considerable interest in the combination of UCH-L1 and phosphorylated forms of TAU [27].

Regarding the distinction of FTD patients from the Control group, our study detected increased levels of the three tested biomarkers—NfL, TAU, and UCH-L1—in the CSF of FTD patients compared to Controls. These findings are in agreement with previous studies reporting increased CSF NfL levels in FTD relative to Controls [49], and the CSF TAU levels of FTD patients are intermediate between Control and AD subjects [3]. Given the heterogeneity of underlying FTD pathologies, a panel of multiple biomarkers specific to different pathologies could be beneficial for distinguishing FTD from other neurological diseases. According to Bolsewig et al., the combination of neuronal pentraxin-2 (NPTX2) (synaptic plasticity), NfL (overall neurodegeneration), and GFAP (astrogliosis) may indicate the complexity of the pathological mechanisms involved in NDs, especially in FTD [49]. Contrary to our study, which did not find any statistical difference between CSF GFAP concentration in FTD patients compared to Control subjects, in the literature, GFAP has been linked with the events of apoptosis and the dystrophy of the astrocytes that have occurred in early stages of the FTD pathology [24]. Despite this fact, our ROC curve analyses highlighted an excellent diagnostic performance of CSF UCH-L1 and NfL in differentiating FTD from Controls (AUC: 0.91 and 0.85, respectively).

Importantly, our data highlight the potential of CSF TAU and, to a lesser extent, CSF GFAP in the differential diagnosis of AD and FTD. In both cases, a statistically significant increase in CSF TAU and GFAP was observed in AD cases compared to FTD in accordance with similar published research [3]. Our data highlight a strong discrimination power of CSF TAU, as well as its potential exploitation for the differential diagnosis of AD and FTD, as inferred from the ROC curve analyses with AUC: 0.8. On the other hand, CSF NfL did not show significantly altered levels between AD and FTD, in contrast to other studies reporting increased CSF NfL levels in FTD compared to AD; however, low discriminatory efficiency of CSF NfL was suggested by the ROC curve analyses in these studies, with the exception of one reporting fair discrimination power (AUC: 0.736). It is worth mentioning that all these studies have utilized ELISA kits in order to measure the NfL biomarker in CSF samples. On the other hand, in our research, all the biomarker measurements were facilitated with the SIMOA technique, which has a 126-fold-higher sensitivity than ELISA [3,50].

Although CSF markers have greater diagnostic accuracy, CSF collection is a highly invasive procedure for the patient and has a number of side-effects in contrast to blood sample collection. Thus, increasing efforts have been made to identify biomarkers in more accessible biological matrices, including urine, saliva, and blood [51,52]. The SIMOA platform is a novel method to study biomarkers of neurodegeneration in plasma, which makes the biomarker search much more feasible than a lumbar puncture [53].

Previously published studies have highlighted the potential use of blood GFAP as an auxiliary marker in the differential diagnosis of AD and FTD patients, reporting increased serum GFAP levels in AD compared to FTD, and discriminatory power characterized by AUC values in the range of 0.65–0.85 [25,54,55,56,57,58]. Regarding NfL or TAU in AD and FTD cases, previous studies have already reported statistically significantly increased levels of plasma NfL in FTD patients compared to AD, with AUC values in the range of 0.61–0.79 [25,54,55,56,57,58,59]. Potential differences between studies may depend on the composition of the patient cohort, the detection method, or the type of analyses performed. Thus, a future differential diagnosis via the SIMOA multiplex panel using plasma AD, FTD, and Control samples would have interesting data to reveal.

Our subsequent goal was to further stratify the samples through the identification of different cohort clusters based on combinations of biomarkers (new and already established) and demographic–clinical characteristics wherever possible. More specifically, we applied the classification performance of the sPLS-DA model in our dimensional dataset to identify the most relevant variables for efficient cohort clustering. We aimed to analyze our data using the sPLS-DA integrative algorithm, combining different types of assay data (CSF, plasma tests), including clinical characteristic data (MMSE, FAB, 5 word recall, CLOX2, Sheltens L, Sheltens R) and demographic data (age, gender, disease duration), to strengthen our clustering of CSF AD and FTD patients. This approach permitted the use of all available data towards the identification of confident disease clusters, strengthening the discriminatory properties of our studied CSF SIMOA markers. It is concluded that positively correlated variables are gender, disease duration, FAB, CLOX2, MMSE, and 5 word recall, while negatively correlated variables are SIMOA GFAP in plasma, SIMOA GFAP in CSF, SIMOA TAU in CSF, ELISA total-TAU in CSF, and ELISA ph-TAU in CSF. The final sample plot of sPLS-DA showed that the subgroup of Controls has a relatively higher correlation with Ab40, Ab42, gender, disease duration, FAB, CLOX2, MMSE, and 5 word recall variables. The AD subgroup preserves a higher correlation with all SIMOA biomarkers (GFAP, NfL, TAU, and UCH-L1), ELISA biomarkers, total-TAU and ph-TAU, Sheltens L&R, and age. Finally, the FTD group has higher correlation with some variables belonging to the other two groups, indicating the need for more-representative samples and measurements.

This is a report on the detection of a multiplex biomarker in a CSF cohort of clinically well-defined AD, FTD, and Control subjects, combining a state-of-the-art, ultrasensitive techniques such as SIMOA. Our study additionally presents the value of new analytical tools, such as sPLS-DA, to facilitate the integration of different sets of variables (in this case, biomarkers and demographic–clinical characteristics), accounting for the maximum variation present in the dataset. This approach has certain limitations regarding the use of mainly non-neurodegenerative neurological cases, the small number of healthy Controls, and the limited clinical characteristics of the Control group. Preferably, a Control group should involve cognitively normal subjects without neurological comorbidities. Regarding the non-neurodegenerative neurological cases, they have received a general CSF diagnostic setup from the physicians. These samples have no inflammation, no blood brain disturbance, no cognitive impairment, or dementia, and have been diagnosed with non-primary neurodegenerative neurological and psychiatric conditions according to the acknowledged standard for neurological, clinical, and para-clinical tests at the time of sampling. However, it is possible, although highly unlikely, that we may have included asymptomatic subjects with underlying AD or FTD pathology in the preclinical stage.

Future research directions will integrate new CSF and/or blood biomarkers into clinical practice, providing a more personalized approach. Even though the performed CSF assay is commercially available and well-validated, large longitudinal studies are needed in order to better determine the cut-off for the positivity, sensitivity, and specificity of a panel of SIMOA biomarkers for efficient early preclinical diagnosis, progression, and response to therapy. The implementation of novel analytical tools such as sPLS-DA will facilitate efficient utilization of all collected data, giving new interpretations and approaches to the diagnosis of neurodegenerative disorders.

## 5. Conclusions

In this study, the primary goal was to assess the diagnostic performance of a panel of four biomarkers—GFAP, NfL, TAU, and UCH-L1—in CSF samples across a clinical cohort of AD and FTD patients and Control subjects using the SIMOA platform. Moreover, we further stratified the samples through the identification of different cohort clusters, based on combinations of biomarkers (new and already established) and demographic–clinical characteristics. Specifically, we applied the classification performance of the sPLS-DA model in our dimensional dataset to identify the most relevant variables to succeed cohort clustering. Our results indicated GFAP and TAU as the most valuable markers for the discrimination of AD from FTD patients; NfL, TAU and UCH-L1 were most valuable in the differentiation of FTD patients from Controls; and lastly, the four of them were statistically significant in terms of the discrimination of AD patients from the Control group. Regarding the sPLS-DA results, the analysis showed that the subgroup of Controls has relatively higher correlation for demographic–clinical characteristics, while the AD subgroup preserves higher correlation for the biomarkers. Finally, the FTD group has a higher correlation with some variables belonging to the other two groups. The results indicate the need for more representative samples, variables, and measurements to train this sPLS-DA model that will be used to classify the dimensional dataset into confidently predefined disease clusters.

## Figures and Tables

**Table 1:** Patient demographics, clinical data, and CSF biomarker concentrations as determined by SIMOA platforms. AD: Alzheimer’s disease dementia; FTD: Frontotemporal dementia; MMSE: mini mental state examination; FAB: frontal assessment battery. All data are presented as median (25th–75th percentile).

	AD *n* = 43	FTD *n* = 33	Controls *n* = 60	*p*-Value
Demographic/clinical data				
Gender (female/male)	20/23	19/14	35/24 *	0.4102 **
Age (year)	65.5 (57.75–76)	62 (56.5–70)	52.9 (31.85–61.7)	<0.0001 ‡
Disease duration (month)	36 (24–48)	36 (24–60)	NA	0.5782 #
MMSE	18 (14–22.25)	25 (16–27.25)	NA	0.0019 #
FAB	7.5 (6–12)	9 (5–13)	NA	0.4078 §
5 word recall	2 (0–3)	4 (2–5)	NA	0.0012 #
CLOX2	8.5 (4–11)	12 (9.5–13.25	NA	0.0040 #
Sheltens L	2 (1–2)	1 (1–2.25)	NA	0.1881 #
Sheltens R	2 (1–2)	1 (0–2.25)	NA	0.7019 #
CSF biomarkers (pg/mL)				
GFAP	9057.82 (5984.45–14,245.77)	5835.62 (2444.36–9526.47)	5353.97 (2976.33–8650.07)	0.0007 †
NfL	1223.64 (965.47–1612.96)	1630.37 (744.51–3261.61)	355.35 (193.43–598.02)	<0.0001 †
TAU	255.5 (180.71–351.01)	124.46 (101.29–194.91)	94.6 (65.05–115.15)	<0.0001 †
UCH-L1	2078.54 (1440.75–4154.26)	2390.84 (1161.55–4037.36)	818.55 (671.92–1003.48)	<0.0001 †

* The rest is unknown; ** χ^2^ test; ‡ ordinary one-way ANOVA; NA: data not available; # Mann–Whitney test; § unpaired *t*-test; † Kruskal–Wallis test.

**Table 2:** Receiver operating characteristic (ROC) curve analysis of CSF GFAP, NfL, TAU, and UCH-L1 to distinguish between clinical patients and Controls by calculating the optimal cut-off points, as well as specificity %, sensitivity %, and its 95% CI and Youden Index (YI).

Controls vs. Disease	Cut Off	Sensitivity %	95% CI	Specificity %	95% CI	YI
UCH-L1: AD vs. Controls	>1260	86.05	72.74% to 93.44%	87.93	77.12% to 94.03%	0.74
UCH-L1: FTD vs. Controls	>1052	84.38	68.25% to 93.14%	79.31	67.23% to 87.75%	0.64
NfL: AD vs. Controls	>688.8	86.05	72.74% to 93.44%	80.70	68.66% to 88.87%	0.67
NfL: FTD vs. Controls	>463.9	87.50	71.93% to 95.03%	73.68	61.02% to 83.35%	0.61
TAU: AD vs. Controls	>137.0	90.70	78.40% to 96.32%	85.96	74.68% to 92.71%	0.77
TAU: FTD vs. Controls	>111.3	69.70	52.66% to 82.62%	70.18	57.34% to 80.47%	0.4
GFAP: AD vs. Controls	>5942	76.74	62.26% to 86.85%	64.29	51.19% to 75.54%	0.41

## Data Availability

The raw data supporting the conclusions of this article will be made available by the corresponding authors on request.

## Supplementary Materials

The following supporting information can be downloaded at https://www.mdpi.com/article/10.3390/biomedicines12061253/s1, Additional biomarkers data: Table S1: Patient CSF and plasma biomarker concentrations, determined by ELISA and SIMOA platforms, respectively. AD: Alzheimer’s disease dementia; FTD: Frontotemporal dementia. All data are presented as median (25th–75th percentile); Table S2: The correlation of each variable with the latent components 1 and 2; Figure S1: Heat Map of the correlation matrix of each variable with the two latent components 1 and 2, where the positive relationship is shown with blue color, the negative with orange, and no relationship with white.
