# Supplementary material for: SIMOA Diagnostics on Alzheimer’s Disease and Frontotemporal Dementia
Source: Biomedicines. 2024 Jun 4;12(6):1253. doi: 10.3390/biomedicines12061253 (PMC11201638; doi:10.3390/biomedicines12061253)
Supplement: Supplementary file 1 [file biomedicines-12-01253-s001.zip › biomedicines-3011844-supplementary.pdf]

Supplementary Files

Additional biomarker data included in sPLS-DA

We included in the sPLS-DA study concentration of ELISA-measured biomarkers (Ab42, Ab40, total-TAU, ph-TAU) in CSF and SIMOA measured biomarkers (GFAP, NfL, TAU, and UCH-L1) in plasma of AD and FTD patients (AD: 43 patients, FTD: 33 patients). The data are summarized in Table S1.

CSF biomarkers Ab42, Ab40, total Tau, and ph-Tau-181 were measured in duplicate with ELISA by commercially available kits (EUROIMMUN Beta-Amyloid (1–42) ELISA; EUROIMMUN Beta-Amyloid (1–40) ELISA; EUROIMMUN Total-Tau ELISA; EUROIMMUN pTau (181) ELISA respectively), according to the manufacturer instructions. Both internal and external quality control measures were utilized to ensure the accuracy of measurements longitudinally. Specifically, a pooled CSF sample was used in every test run, as internal control, resulting in an over >90% between-run precision. Samples provided by "The Alzheimer's Association's QC program", were utilized as additional external pooled CSF samples for validating results reliability regardless of the kit's lot number.

GFAP, UCH-L1, NfL and Tau concentrations in the plasma were determined using a commercial assay kit [Neuro 4 plex, Quanterix (Product number: 103345)] that has already been optimized for certain marker proteins and measured in the SIMOA-SR-X instrument (Quanterix, Billerica, MA, United States). Analysis was performed according to the manufacturer instructions. Plasma samples were diluted 4x to a total volume of 100 μL. Two internal controls with a defined protein concentration were included in the assay and data were subjected to further analysis only when both internal assay controls were within the expected range (less than 10% variation).

Table S1. Patient CSF and plasma biomarker concentrations, determined by ELISA and SIMOA platforms, respectively. AD: Alzheimer's disease dementia; FTD: Frontotemporal dementia. All data are presented as median (25th – 75th percentile).

	**AD n=43**	**FTD n=33**
CSF ELISA biomarkers (pg/mL)		
Ab 42	365.3 (300.23-476.95)	574.7 (423.75-842.48)
Ab 40	6439.35 (4986.43-8990.15)	4713.9 (4168.3-7158.5)
total-TAU	574.4 (452.03-767.25)	310.5 (218.9-408.9)
ph-TAU	124.25 (102.05-155.1)	33.6 (27.4-41.6)
PLASMA SIMOA biomarkers (pg/mL)		
GFAP	205.15 (125.38-274.6)	131.06 (76.8-170.58)
NfL	11.77 (9.08-18.95)	17.35 (10.66-29.02)
TAU	3.36 (2.3-4.21)	3.61 (2.87-4.78)
UCH-L1	11.03 (6.25-25.33)	23.11 (10.3-34.66)

Heat Map of the correlation matrix of each variable with the two latent components 1 & 2

We included a table containing the correlation of each variable with the latent components 1 and 2 of the sPLS-DA, where the values range from -1 to 1. Values close to 1 indicate a strong positive relationship, values close to -1 indicate a strong negative relationship, and values around 0 indicate no relationship (Table S2).

Table S2. The correlation of each variable with the latent components 1 and 2.

Correlation of each variable with component 1		Correlation of each variable with component 2	
UCHL1_CSF	-0.463510853	UCHL1_PLASMA	0.41525038
UCHL1_PLASMA	-0.447622249	ph_tau	-0.37980474
TAU_CSF	-0.409652596	NFL_CSF	0.30705427
Age	-0.345623111	UCHL1_CSF	0.27103864
Ab42	0.288361394	Ab40	-0.25852026
t_tau	-0.263525525	TAU_CSF	-0.25568589
ph_tau	-0.221294785	X5word_recall	0.24695715
GFAP_CSF	-0.188793037	GFAP_CSF	-0.22861262
NFL_CSF	-0.120103274	NFL_PLASMA	0.22130323
X5word_recall	0.089922221	clox2	0.21217393
Ab40	0.087202162	GFAP_PLASMA	-0.20256014
Gender	0.084491576	MMSE	0.20086237
GFAP_PLASMA	-0.082772112	t_tau	-0.19090486
MMSE	0.074354489	TAU_PLASMA	0.13152401
clox2	0.067015748	disease_duration	0.13116032
disease_duration	0.057696208	Age	0.10191031
Sheltens_L	-0.032822851	Gender	0.08088425
FAB	0.027594952	FAB	0.06676140
NFL_PLASMA	0.027049923	Sheltens_L	-0.03788193
Sheltens_R	-0.016542920	Ab42	-0.02390856
TAU_PLASMA	0.003380857	Sheltens_R	0.02011167

Moreover, we depicted this correlation matrix into a Heat Map where the positive relationship is shown with blue color, the negative with orange and no relationship with white Figure S1. These results are in complete agreement with Figure 3(a), where positively correlated variables with components 1 & 2 i.e. x5 word recall, MMSE, clox 2, disease duration and FAB are shown with blue color on Heat Map, while negative correlated variables with components 1 & 2 i.e. GFAP_plasma, GFAP_CSF, TAU_CSF, total-TAU and ph-TAU are shown with orange color on Heat Map.

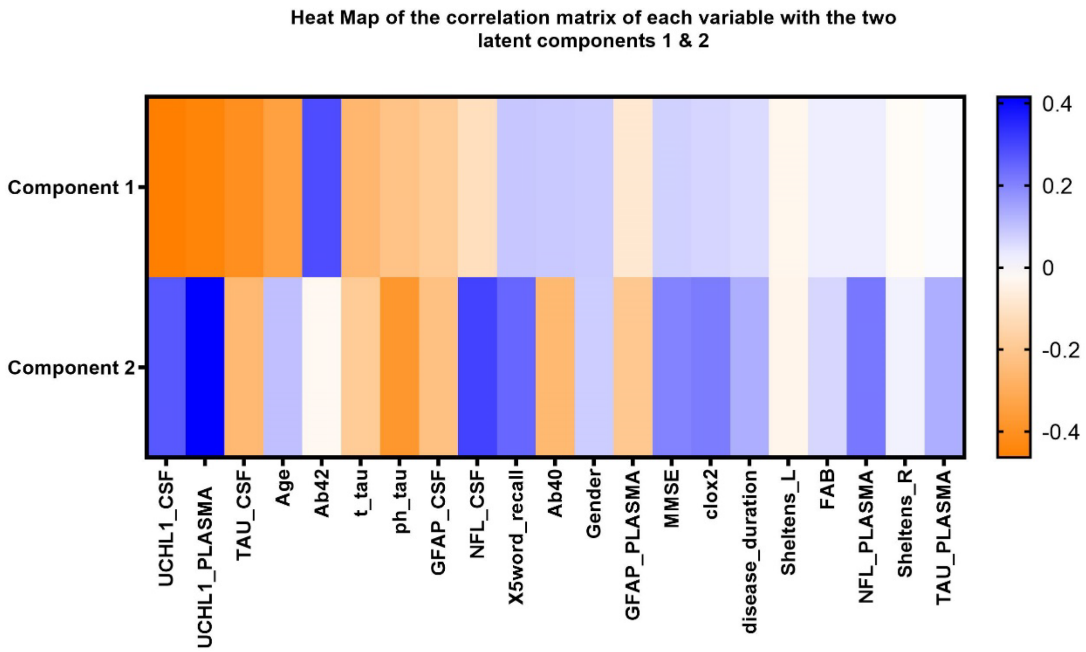

Figure S1. Heat Map of the correlation matrix of each variable with the two latent components 1 & 2, where the positive relationship is shown with blue color, the negative with orange and no relationship with white.
